# Supplementary material for: Sensory discrimination of chemical and temperature stimuli in the acoel Symsagittifera roscoffensis
Source: PLoS One. 2025 Aug 21;20(8):e0330757. doi: 10.1371/journal.pone.0330757 (PMC12370049; doi:10.1371/journal.pone.0330757)
Supplement: Attachment — Submitted filename: review_Komarov_2025.pdf [file pone.0330757.s002.pdf]

General comments

In this paper, the authors investigated the chemosensory (salinity, amino acids and algae) and thermosensory (temperature) capacity of wild-caught acoel *Symsagittifera roscoffensis* in a laboratory setting. This is an exploratory study to determine whether these worms are able to detect and respond to these different stimuli. The results show that Symsagittifera roscoffensis is able to detect both chemical and physical cues, but at different levels. Only the amino acid Trytophan initiated a significant response. Only the 68 ppm salt concentration initiated a significant response. According to a following test, the initiated response seems to be aversion. For algae, F/2 medium seems to have elicited a preference response, while *T. convolutae*, *C. concordia* and *T. chuii* an avoidance response. According to a following test, the initiated response for the symbiont *T. convolutae* seems to be aversion. Finally, only the presence of high temperature elicited a response from the worms. In all cases, the worms seemed to avoid high temperatures.

Chemical and sensory ecology are important and growing fields, and I'm delighted to see the authors' interest in these still little-explored research fields. This exploratory study will help expand knowledge in these two fields, which is particularly important in the context of climate change, where aquatic environments are particularly affected. The study system chosen is very interesting and original. I applaud the authors' boldness in focusing on a lesser-known group of animals that are nonetheless the foundation of trophic webs.

However, I have a few issues that need to be addressed before publication. I also offer suggestions for improving the manuscript.

*Next time, I strongly recommend to include line numbers to facilitate communication.

Major issues

The experiments carried out seem satisfying. However, the manuscript lacks scientific rigor. Several corrections need to be applied to meet publishable scientific quality standards.

1. The problematics/research question, aims, hypotheses, method and results should all be clearly aligned. At present, it is difficult to understand the study before reading the results. I recommend restructuring the ideas and making sure that the method (protocol) and results address the research question and objectives. A clear statement of whether the objectives have been met and whether the hypotheses are supported by the results should also be present. Without restricting authors to the classic writing format, the minimum requirements for assessing the validity of a study must be met.

2. The authors mention that ASW (34ppm) is aversive. However, this is not what is measured in the experiment. It's possible that the worms simply prefer the non-salty side without necessarily avoiding the other. I suggest that the authors add some nuance to their statements and don't mix up the results with interpretation. The PI (result) indicates that more worms chose the non-salty side. The interpretation is that the salty side is aversive

for the worms (or that the non-salty side is prefered). Preference/avoidance is an interpretation of the results. This interpretation should therefore be accompanied by a "We suggest/propose that...". This is repeated for the presentation of each result. Since the results seem to be combined with the discussion, authors should check each result presented to ensure that the difference between the result and the interpretation is unambiguous.

3. The discussion lacks references. Some paragraphs have 0 to 1 references. With less than 2 or 3 sources, it's difficult to have a deep analysis of the situation. It would be useful to use a variety of references and examples to deepen the ideas. If there are no references on this species, which seems understudied according to the authors, the examples may come from similar species or those living in similar environments.

   The findings should also be put into a broad context. Does it support existing literature or not? If not, why not?

4. The discussion should contain the limits/caveat of the experiment. Scientific rigor requires the assessment of limits. The authors should describe some shortcomings that may affect data interpretation, e.g. :

   What are the confounding factors? What are the differences between the laboratory and the natural environment? What could have been improved? What should be tested to verify interpretations? What lacked precision?

   Without invalidating their study, the authors must discuss certain points, as no study is perfect.

5. The method is missing a section on statistical analysis. Without this section, it is not possible to assess the validity and reliability of the results and conclusions drawn by the authors. I will, however, make a few comments based on what is mentioned in the figure titles.

   a. Was Dunnet's T3 multiple comparisons test carried out for each time (2, 5, 10 min) separately?
   b. Why did the authors choose these tests? Were the application conditions respected? These details should be included in the method.
   c. Did a single worm do several trials (tyrosine, glutamine, etc.)? If so, were the worms always in the same group? If the groups remain the same, it should be added to the model as a random factor since the analyses are based on repeated measures (One-way repeated measures ANOVA or GLMM).

Minor issues

6. I suggest that authors proofread the entire text to correct spelling, grammatical and wording errors. To facilitate correction, authors can read the text starting from the end, or ask a third party who was not involved in drafting to read the text. In addition, certain scientific words relating to animal behaviour are not used correctly. Authors need to revise certain definitions and ensure that the scientific terms used support exactly what they mean.

7. Authors will have to modify the formatting of references to meet the journal's requirements :
References are listed at the end of the manuscript and numbered in the order that they appear in the text. In the text, cite the reference number in square brackets (e.g., "We used the techniques developed by our colleagues [19] to analyze the data"). PLOS uses the numbered citation (citation-sequence) method and first six authors, et al.
https://journals.plos.org/plosone/s/submission-guidelines#loc-introduction

Abstract (page 2)

8. In the second sentence, the authors mention that "understanding how distinct sensory cues elicit or modulate certain behaviour thus provides insights into the neuronal adaptations to rapid and continuous changes in the surrounding world". However, there is no reference to neuronal adaptation afterwards, although this seems to be an important subject of the study they way it is mentionned. I consider this paper to be in the field of animal behaviour rather than neuroscience. Authors should be careful about the scope of their claims.

9. When stating the general problem (lack of knowledge on other sensory systems), I recommend giving a short explanation on why bridging the gap matters, to give more context and justify the purpose of the study.

10. While keeping this section concise with the main result, I suggest going into a little more detail. The method and main results should be incorporated to quickly understand the study when first reading the abstract. I suggest integrating the approach and the main results in a single sentence (e.g. Using XXX method, we found YYY).

11. What does the main result reveals? The aim of the study seems to be to explore the yet unknown sensory systems of *S. roscoffensis*. What do the results reveal about this? Does it suggest that *S. roscoffensis* can detect chemical and thermal cues?

    I suggest adding a final sentence or two about the wider context of the results. For example, but without limitation, authors could mention:
    -What the results demonstrate;
    -What are the advances/implications in relation to previous works;
    - How does the results change the understanding of this field (sensory ecology or neuronal adaptations) / how significant are the results?

Since these additions are likely to increase the size of the abstract, I suggest cutting into the study backgroud, which currently takes up a lot of space compared to the results (i.e. everything before "Currently little is known about...").

12. "is" should be changed for "are" in the following sentence : "When the acoel *Symsagittifera roscoffensis* is exposed…"

13. The study topic is truly interesting and captivating. I think it's worth extending the introduction a little to take the time to properly introduce the concepts and justify the aim of the study.

Introduction

14. **Page 3 :** The first paragraph of the introduction should include at least one or two references. An interesting way of inserting them is to add examples to support the facts.

15. **Page 3 :** In the first paragraph, the authors mention that environmental signals elicit or modulate a behaviour. I suggest to change "behaviour" for "response" or to add nuance (such as "can elicit") as it can be a behavioural or physiological response.

16. Through the manuscript, the authors alternate between the terms "signal" and "cue". Authors should be careful to use the correct term, as the two do not have the same meaning. A signal provides intentional (active) information with the aim of influencing the receiver's behaviour. Signals are actively produce to communicate. A cue provides unintentional (passive) information, which may be environmental or behavioural (Freeberg et al., 2021)

17. **Page 3 :** In the first sentence of the second paragraph, "thus" should be removed : "The highly variable environmental physical and chemical conditions of intertidal ecosystems thus likely…"

18. **Page 3 :** For precision, I suggest that the authors define what they mean by intertidal ecosystems.

19. **Page 3 :** "…the direct exposure to light also provides advantages for certain physiological adaptations." The authors should be careful with the wording. It's not the environment that offers an advantage to an adaptation, but the opposite.

20. **Page 3 :** "*S. roscoffensis* challenges some conventional metabolic principles...". Is it possible to name a few as examples as "metabolic principles" is rather vague.

21. **Page 3 :** The following sentence is unclear : "However, despite receiving a range of nutrients and energy from its symbiont (Selosse, 2000), little is known about whether S. roscoffensis requires additional external nutrients (possible mixotrophy) or cues to thrive in this changing environment; and if so, how a hypothetical chemosensory system is used to forage for them."

I understand that the authors question the possible use of additional nutrients and additionnal cues. If this is the case, there has been no mention yet of using any kind of cue. At the beginning of the paragraph, exposure to direct light was discussed, but no clear link was made with the worm and its symbiosis with an algae.

I suggest changing the order of presentation of the concepts in the paragraph to clarify the idea supported.

22. **Page 4 :** The following sentence is unclear : "While lacking obvious or canonical chemosensory organs (only candidates: (Bery et al., 2010; Sakagami et al., 2024; Todt andTyler, 2007)), *S. roscoffensis* possesses light-sensitive rhabdomeric eyes and a gravity-sensing statocyst (Bailly et al., 2014; Lanfranchi, 1990; Sprecher et al., 2015; Yamasu, 1991)."

    What are obvious and canonical chemosensory organs? I think that some examples or a definition would be beneficial to understanding. In the same vein, authors should include a definition or the utility of light-sensitive rhabdomeric eyes and gravity-sensing statocyst.

23. **Page 4 :** As with the abstract, the introduction misses details on the importance of bridging the knowledge gap. Why should we know more about the sensory system of *S. roscoffensis*? Why is this information important to the authors' field of study?

24. **Page 4 :** "and" should be changed for "in", and the comma between interdidal and environment should be removed in the following sentence : "Given that this worm lives and aquatic, intertidal, environment…".

25. **Page 4 :** The authors hypothesize that *S. roscoffensis* should be able to respond to chemical cues from their symbiont and free-living relatives. This is the first mention of cues emitted by the symbiont. This fact should have been mentioned before. I suggest that authors make sure the story told in the introduction leads to the problematics to be investigated and to the aim, and that all this is clearly related to the hypotheses. The research problem/question, hypotheses and predictions must be coherent and aligned.

Results

26. Authors must choose their spelling of behaviour. Sometimes it is spelled with a "u" and sometimes without.

27. **Page 5 :** This sentence is unclear : "Other neurotransmitter AAs such as glycine, or neurotransmitter
    precursor AAs such as tyrosine (dopamine and noradrenaline precursors), tryptophan (serotonin precursor), were not tested for in previous studies."

    Was not tested for what?

28. The verb "to sense" if often use (e.g. : " Hereby, we tested whether S. roscoffensis is able to sense and respond to the presence of these AA"). The use of this term seems incorrect as it refers to a feeling. I suggest replacing it with the verb "to detect".

29. The link between the use and the detection of AAs is unclear to me. What use is it to worms to detect AAs that can act as neurotransmitters? Is it an essential nutrient that should be present in their diet? If it's the symbiont that produces AAs for the worm, why should the worm be able to detect them in the environment? Is the aim of this experiment to see whether worms can detect AAs or to check whether glycine, tyrosine and tryptophan are used by worms?

30. The authors alternate between AA and amino acids. The use of one or the other must be constant.

31. The presentation of the results is not indicative (e.g. PI=-0.1, p=0.74). Many details are missing. These details reinforce the quality and rigor of the results, making the study much more robust.

    a. Since the method isn't presented first, (PI) should be placed in brackets as soon as *preference indices* is first mentioned in the text. It should also be mentioned that the PI value corresponds to the mean PI (from what I understood).
    b. I recommend keeping the PI value and the statistical test separate, as they are two different things. The PI should be accompanied by its ± SEM or SD (mean = -0.001, SEM = 0.002) or (mean ± SEM : -0.001 ± 0.002)
    c. The test from which these results come must be mentioned.
    d. The test statistic and its value, the degrees of freedom (or sample size, according to the test) and the p-value must be mentioned. The p-value means nothing without the value of the test statistics and the ± SEM or SD. I recommend reading Amrhein et al. 2019.

    Following these suggestions, the presentation would look like this, depending on the statistical test used :
    (PI = -0.001, SEM = XX; Brown-Forsythe ANOVA : $F_{df1, df2}$ = XX, p = 0.9996)

    Authors should also ensure that the presentation of results is consistent. Several variants are currently used :
    (10 min,PI=-0.22, p=0.09)
    (mean preference index -0.45, p<0.0001 vs control)
    (PI=-0.001, p=0.9996)

32. The use of the terms timepoint and timescale should be consistent, with only one of them retained throughout the text. X-minute timepoint is more precise. By using the latter, it is no longer necessary to mention the time in brackets with the results.

33. The authors should be careful with the wording of this sentence, there are many unfinished ideas :

Page 6 : Considering the dynamic nature with varying salinity in intertidal waters due to evaporation and temperature changes during low tides, it has been shown that during low tides the salinity of sea water can change by up to 300% (Geng et al., 2016), we then asked whether changes in salinity of the substrate would result in a preference phenotype.

What is a preference phenotype? This seems a bit out of nowhere as the main goal of the study is to investigate the chemosensory and thermosensory sensitivity of *S. roscoffensis*. I suggest defining the term and making a clearer link with the main objective of the study.

34. **Page 7 :** For the barrier-assay, the authors should present the results in the same way as before, i.e. the average percentage of crossing with the error, and the result of the statistical tests. Idem for algae tests.

35. **Page 7 :** For the barrier-assay, how the authors determined that this was an aversion and not some other physiological effect? What would have been the observed results if the cause of non-crossing was, for example, reduced mobility?

36. **Page 8 :** "These experiments support the notion that the animals…". "Animal" should be changed for the species name. This should be done throughout the text to ensure clarity.

37. **Page 8 :** "This in turn suggests that while low temperatures are neither attractive nor aversive, high temperature is decidedly an aversive cue." Why can't it be a preference for lower temperatures rather than an avoidance of higher ones? How did the authors come to this interpretation?

Discussion

38. **Page 9 :** "Here we analysed the preferences to chemical and physical cues of the acoel flatworm Symsagittifera roscoffensis…" The aim in the introduction should be the same as the one mentioned in the discussion.

39. Is the aim achieved? Do the results support the hypotheses? To me, these two elements are essential and and fundamental to rigorous scientific work. Yet this is missing.

40. **Page 9 :** "it is logical that the animals would prefer a specific level of salinity to remain in an isotonic environment and avoid a hypotonic one."

    Why is this logical? If these worms are used to constant and drastic changes, isn't their physiology adapted to this? Wouldn't they be able, by homeostasis for instance, to survive in different salinity levels without needing to move? Are they able to move great distances (I imagine that a movement of 87 mm, the size of the petri, in nature does not really change the salinity of the environment)? Could it be a behaviour related to foraging, reproduction or feeding? Do the authors have any references to back up their claims?

41. **Page 10 :** Could the aversive response be due to the algae taking some control over its host (kind of parasite manupulation, but with a symbiont) (Hughes & Libersat, 2019)?

42. **Page 11 :** "Thus, the "low temperature" condition in our assay may not be sufficiently lower than the temperature experienced by S. roscoffensis in the natural environment." How did the authors select the temperatures and salinity levels? Why not choose the maximum and minimum limits of the natural environment?

43. **Page 11 :** The last sentence is hard to understand and rather vague. I suggest rephrasing and that the link between the final conclusion and the rest of the study be more thoughtful.

Materials and Methods

44. There is a lack of detail in the collection and maintenance section to ensure reproducibility. As experiments are performed on live animals, capture and maintenance conditions may influence the animals' behavioural response measured during trials. I recommend that authors add information about:

    - GPS coordinates at collection site
    - Date of capture (at least month and year)
    - Lifestage or average size, if known
    - Sex (M, F or unknown)
    - Presence or absence of *Tetraselmis convolutae* symbiont
    - Number of worms initially captured, number of worms per plastic container
    - Date of trials (How long they acclimated to the lab?)
    - Relevant holding conditions (e.g. temperature, oxygen level). Were conditions constant or dependent on outside weather conditions? Has the water been treated in any way (UV, sand filter, etc.) to prevent infection or parasites?
    - Feeding regime during holding and trials (I imagine this may influence the preference for one side of the agarose and the motivation for foraging).

45. In order to verify the thinking behind the experimental protocol, I have a few questions. The aim here is not to cast doubt on the experiments, but to explore possible limitations and confounding factors :

    a. Was the number of worms per petri dish too high to allow each worm to move freely and voluntarily and to make a real choice? Could the degree of sociability of these worms have influenced the result? How did authors choose the number of worms per petri dish?

    b. What was the temperature in the petri dish during chemical trials? Was it stable? This is important because temperature preference is tested later.

    c. Was the lighting constant and identical on both sides? Could this have influenced the worms' preference?

d. Did the AAs and algae remain in the agarose gel or gradually dilute in the water? If diluted gradually, could this have influenced the choice over time? How did the authors ensure that the worms could actually detect the cues in the agarose?

e. Were the experimenters present during the trials? If so, did this influence the worms' response?

f. Did counting during trials disturb the worms? What have you done to reduce the disruption caused by counting? Did you count from a video/image after?

g. Did you count manually or with a software? If you counted manually, how did you reduce or limit the biases of the person counting? If you counted with a software, what was its name/brand?

46. *S. roscoffensis* were grouped into groups of 50 to 200 individuals. How many trials (number of group) were carried out for each experiment? This should be mention in the method.

47. Have non-responsive individuals been removed from the count? How many did you remove? What were the criteria for determining that an individual was non-responsive?

48. If not already done, I strongly suggest that authors read Jutfelt et al. (2017) article on two-current choice flumes for testing avoidance and preference in aquatic animals. The experimental apparatus is not quite the same, but I believe that certain ideas could guide the authors in explaining their method.

    Here's a point raised in the article by Jutfelt et al. (2017) : "In any event, sufficient exploration of the arena by the test animal is an obvious prerequisite before attribution of side preferences can occur."

    Did the worms have the opportunity to sample the cues on both sides of the petri dishes before the experiment began? If not, how can the authors be sure that a preference was measured if some worms didn't even know what was on the other side?

49. **Page 15 :** Please correct the names of statistical tests : Brown-Forsythe and Welch ANOCA test with Dinnet's T3 multiple comparisons tested against the control group.

50. **Page 16 :** There's an analysis of the results in the title of figure 3 C, which isn't there for the other figures. I think this can be removed.

51. There is no title for figure 4.

References

Amrhein, V., Greenland, S., & McShane, B. (2019). Scientists rise up against statistical significance. *Nature*, *567*(7748), 305-307. https://doi.org/10.1038/d41586-019-00857-9

Freeberg, T.M., Book, D.L., Jung, H., Kyle, S.C. (2021). Communication, Cues, and Signals. In: Shackelford, T.K., Weekes-Shackelford, V.A. (eds) Encyclopedia of Evolutionary Psychological Science. Springer, Cham. https://doi.org/10.1007/978-3-319-19650-3_2728

Hughes, D. P., & Libersat, F. (2019). Parasite manipulation of host behavior. *Current Biology*, *29*(2), R45-R47. https://doi.org/10.1016/j.cub.2018.12.001

Jutfelt, F., Sundin, J., Raby, G. D., Krång, A., & Clark, T. D. (2017). Two-current choice flumes for testing avoidance and preference in aquatic animals. *Methods in Ecology and Evolution*, *8*(3), 379-390. https://doi.org/10.1111/2041-210X.12668
