# Supplementary material for: Sensory discrimination of chemical and temperature stimuli in the acoel Symsagittifera roscoffensis
Source: PLoS One. 2025 Aug 21;20(8):e0330757. doi: 10.1371/journal.pone.0330757 (PMC12370049; doi:10.1371/journal.pone.0330757)
Supplement: Attachment — Submitted filename: Reviewers response.docx [file pone.0330757.s001.docx]

Reviewers' comments:

Reviewer's Responses to Questions

**Comments to the Author**

1. Is the manuscript technically sound, and do the data support the conclusions?

The manuscript must describe a technically sound piece of scientific research with data that supports the conclusions. Experiments must have been conducted rigorously, with appropriate controls, replication, and sample sizes. The conclusions must be drawn appropriately based on the data presented.

Reviewer #1: Yes

2. Has the statistical analysis been performed appropriately and rigorously?

Reviewer #1: Yes

3. Have the authors made all data underlying the findings in their manuscript fully available?

The [PLOS Data policy](http://track.editorialmanager.com/CL0/http:%2F%2Fwww.plosone.org%2Fstatic%2Fpolicies.action%23sharing/1/010f0196d339517f-69acf3bd-328e-4080-9a49-f9dfeac09180-000000/QFY5o9ezbkkRg3Eqn-3QDI-pdtWCvBXeGJBBpo_UhsE=211) requires authors to make all data underlying the findings described in their manuscript fully available without restriction, with rare exception (please refer to the Data Availability Statement in the manuscript PDF file). The data should be provided as part of the manuscript or its supporting information, or deposited to a public repository. For example, in addition to summary statistics, the data points behind means, medians and variance measures should be available. If there are restrictions on publicly sharing data—e.g. participant privacy or use of data from a third party—those must be specified.

Reviewer #1: Yes

4. Is the manuscript presented in an intelligible fashion and written in standard English?

PLOS ONE does not copyedit accepted manuscripts, so the language in submitted articles must be clear, correct, and unambiguous. Any typographical or grammatical errors should be corrected at revision, so please note any specific errors here.

Reviewer #1: Yes

5. Review Comments to the Author

Please use the space provided to explain your answers to the questions above. You may also include additional comments for the author, including concerns about dual publication, research ethics, or publication ethics. (Please upload your review as an attachment if it exceeds 20,000 characters)

**Reviewer #1:** I have no major comments on this manuscript. The claims are adequately supported by the data, the discussion is reasonably nuanced, and better knowledge of Symsagittifera's sensory preferences is a useful addition to the literature.

**We thank the reviewer for these kind words**

Minor comments:
- Not sure how feasible it is to do additional experiments for this project, so if that isn't possible, it might be worth discussing the negative results in more detail. Specifically: why did you choose 20mM for amino acids? How might the results be different at different concentrations? Might different AAs diffuse through the agarose at different rates?

**Thank you for this comment. Indeed, at the moment it would not be feasible to perform additional experiments, however we believe that in the context of this study the questions are adequately investigated.**

**Regarding amino acid concetnration, we have added the relevant text in the discussion section which reads:**

**The 20mM concentration for amino acids was chosen as it is the standard for small animal behaviour, such as in the *Drosophila* larva, though it has been used in vertebrates as an average concentration where they are responsible (for instance in spider monkeys) References are added to the text. It has been shown to provide sufficient responses without being toxic. While higher than the physiological concentrations or ones found in regular food, the diffusion through agarose in general is slower than in water. We do not believe that diffusion rates will differ between different amino acids, as the average size is around 4Å, while agarose gel pores typically range from between 30-200nM. Other factors, such as ion concentrations may be more relevant, thus for our experiments concentrations were kept equal.**

- Along similar lines, perhaps the concentrations of the symbionts were too high, causing an aversive response that might be context-dependent?

**These concentrations of symbionts are the same that we are using in the laboratory (in fact, in the laboratory of Xavier Bailly) to maintain stable cultures of S. roscoffensis, able to close their life cycle. They follow similar concentrations used by Provasoli and Douglas in the past (see next comment). In this context we would call those concentrations “physiological”, in the sense that they work well and don’t produce any disruptive response.**

- You speculate that immature animals may find the symbionts attractive. Is it possible to do this experiment?
- Perhaps it would help to add some context to the discussion - eg by explicitly comparing your results to expectations or relevant data from previous work, especially Thomas et al. 2023

**-Correct. This experiment has been done in different laboratories, where the algae has been added to the aposymbiotic animals (hatchlings). Provasoli et al. (1968), Douglas (1983) and Arboleda et al (2018) have done, and reported, the positive results of this experiments. One of us, Dr. Xavier Bailly, has developed stable cultures of S. roscoffensis and demonstrated (unpublished, though) that these algae+host mixtures in culture are perfectly viable and hasn’t observed any toxic effect. In fact, the possibility of mixing algae and aposymbiotic hosts allowed him to maintain cultures for three years in a row (without any problem).**

**-Our work represents a good complement to the one published by Thomas and collaborators, where they test the response of S. roscoffensis to several physical and chemical cues. Though they use the same species, their stimuli are different than those studied by us (light, vibration, sea water or frozen worms). This complementary approach has now been stressed in the text. Perhaps the most relevant issue here, which merits further experimentation, is the response to a frozen worm versus a worm-extract. Our negative response might be the result of chemicals extracted from the animals during the maceration process. It is possible that their repellent nature would be a natural response of worms in a situation where the environment signals stress or mortality to the living specimens, thus generating a flight response from the “mortality cues”.**

6. PLOS authors have the option to publish the peer review history of their article ([what does this mean?](https://track.editorialmanager.com/CL0/https:%2F%2Fjournals.plos.org%2Fplosone%2Fs%2Feditorial-and-peer-review-process%23loc-peer-review-history/1/010f0196d339517f-69acf3bd-328e-4080-9a49-f9dfeac09180-000000/dsATG4hwmyOoosUXIXceZY_kNZnYDs3ymi_JruwdUlg=211)). If published, this will include your full peer review and any attached files.


If you choose “no”, your identity will remain anonymous but your review may still be made public.


**Do you want your identity to be public for this peer review?** For information about this choice, including consent withdrawal, please see our [Privacy Policy](https://track.editorialmanager.com/CL0/https:%2F%2Fwww.plos.org%2Fprivacy-policy/1/010f0196d339517f-69acf3bd-328e-4080-9a49-f9dfeac09180-000000/3P_f9t81dIWfTIEn0GFKwK8amTUITYMqGKbUXnK-yZc=211).

Reviewer #1: No
