# Supplementary material for: Sensory discrimination of chemical and temperature stimuli in the acoel Symsagittifera roscoffensis
Source: PLoS One. 2025 Aug 21;20(8):e0330757. doi: 10.1371/journal.pone.0330757 (PMC12370049; doi:10.1371/journal.pone.0330757)
Supplement: Attachment — Submitted filename: Rebuttal 2.docx [file pone.0330757.s003.docx]

We acknowledge the referees for their invaluable input towards improving this manuscript. Their suggestions have undoubtedly elevated the original manuscript to be more thorough, accurate, and informative. Thank you.

**General comments**

In this paper, the authors investigated the chemosensory (salinity, amino acids and algae) and thermosensory (temperature) capacity of wild-caught acoel *Symsagittifera roscoffensis* in a laboratory setting. This is an exploratory study to determine whether these worms are able to detect and respond to these different stimuli. The results show that Symsagittifera roscoffensis is able to detect both chemical and physical cues, but at different levels. Only the amino acid Trytophan initiated a significant response. Only the 68 ppm salt concentration initiated a significant response. According to a following test, the initiated response seems to be aversion. For algae, F/2 medium seems to have elicited a preference response, while *T. convolutae*, *C. concordia* and *T. chuii* an avoidance response. According to a following test, the initiated response for the symbiont *T. convolutae* seems to be aversion. Finally, only the presence of high temperature elicited a response from the worms. In all cases, the worms seemed to avoid high temperatures.

Chemical and sensory ecology are important and growing fields, and I'm delighted to see the authors' interest in these still little-explored research fields. This exploratory study will help expand knowledge in these two fields, which is particularly important in the context of climate change, where aquatic environments are particularly affected. The study system chosen is very interesting and original. I applaud the authors' boldness in focusing on a lesser-known group of animals that are nonetheless the foundation of trophic webs.

However, I have a few issues that need to be addressed before publication. I also offer suggestions for improving the manuscript.

*Next time, I strongly recommend to include line numbers to facilitate communication.

Thank you for this suggestion, we have included line numbers in this revised version. The line numbers present in this response are in relation to the “track changes” version of the manuscript.

**Major issues**

The experiments carried out seem satisfying. However, the manuscript lacks scientific rigor. Several corrections need to be applied to meet publishable scientific quality standards.

1. The problematics/research question, aims, hypotheses, method and results should all be clearly aligned. At present, it is difficult to understand the study before reading the results. I recommend restructuring the ideas and making sure that the method (protocol) and results address the research question and objectives. A clear statement of whether the objectives have been met and whether the hypotheses are supported by the results should also be present. Without restricting authors to the classic writing format, the minimum requirements for assessing the validity of a study must be met.

Thanks for the suggestion. Yes, we are aware that the introductory paragraph was, most probably, unclear and lacking a proper explanation of the study context. We have added the following paragraph for clarity:

*“The phylogenetic position of acoels, along with the fact that some species live in close association with algae, has made them of particular interest in the fields of evolutionary developmental biology (evo-devo) and comparative physiology. However, most existing studies on these animals focus on developmental and anatomical aspects, with relatively few addressing their physiology or the influence of environmental conditions on behavior, survival, or the dynamics of the host–algae association. To address this gap, we initiated a series of experiments aimed at testing the effects of various chemicals on these animals. Most of the compounds we tested—such as variations in salinity and temperature—are highly relevant to the rapidly changing conditions in coastal environments, where many acoel species naturally occur.”*

1. The authors mention that ASW (34ppm) is aversive. However, this is not what is measured in the experiment. It's possible that the worms simply prefer the non-salty side without necessarily avoiding the other. I suggest that the authors add some nuance to their statements and don't mix up the results with interpretation. The PI (result) indicates that more worms chose the non-salty side. The interpretation is that the salty side is aversive

We thank the reviewer for pointing out this lack of clarity. We have thus adjusted the results section involving these experiments to read as follows (Lines 166-167): “The finding that S. Roscoffensis appears to prefer lower salt concentrations suggests that this condition is preferential for the acoel”

We would like to point out, however, that the ‘aversion’ was further determined by the subsequent barrier assay experiments, where high salinity was able to negate the positive phototaxis behaviour

1 -salty side is prefered). Preference/avoidance is an interpretation of the results. This interpretation should therefore be accompanied by a "We suggest/propose that...". This is repeated for the presentation of each result. Since the results seem to be combined with the discussion, authors should check each result presented to ensure that the difference between the result and the interpretation is unambiguous.

We thank the reviewer for pointing this out. Indeed, the interpretation may appear somewhat liberal. We have adjusted the text in the following places to remedy this:

Line 133: ...largely match those of the plain agarose control, suggesting animals did not prefer either side

Line 138: we observed that there was a moderate preference across the longer timescale

Line 154: We observed one-fold ASW (34ppm) showed a mildly positive, albeit insignificant, preference index

Line 158: we observed that the PI for two-fold ASW concentration (68ppm) was significantly negative across the time series

Line 159: However, they show a slight but statistically insignificant avoidance negative PI of to T. striata extracts

Line 213: No difference in the side preference index

Line 215: no significant preference index

Line 218-219: S. roscoffensis the animals tend to prefer show a positive PI to the low temperature side

1. The discussion lacks references. Some paragraphs have 0 to 1 references. With less than 2 or 3 sources, it's difficult to have a deep analysis of the situation. It would be useful to use a variety of references and examples to deepen the ideas. If there are no references on this species, which seems understudied according to the authors, the examples may come from similar species or those living in similar environments.

We agree with the reviewer that the number of references is sparse, however this is, indeed, due to a severe lack of modern (<50 years) research conducted on this topic, in this scenario, in marine animals. We have now included, to our knowledge, all relevant references that are available, and in turn focused the discussion on exploring the potential significance of our results

The findings should also be put into a broad context. Does it support existing literature or not? If not, why not?

1. The discussion should contain the limits/caveat of the experiment. Scientific rigor requires the assessment of limits. The authors should describe some shortcomings that may affect data interpretation, e.g. :

What are the confounding factors? What are the differences between the laboratory and the natural environment? What could have been improved? What should be tested to verify interpretations? What lacked precision?

Thank you for pointing this out. We have adjusted the discussion section in order to address the reviewer’s concerns:

Line 347: Under laboratory conditions, i.e. agarose substrate vs natural sand, our findings indicate...

Lines 367-378: To isolate responses exclusively to the stimuli of interest, our experimental conditions therefore differ from the natural environment in which S. roscoffensis is typically found. For instance, the substrate used in our experiment was agarose, as opposed to the natural substrate of sand, which may contain a large variety of chemical and biological contaminants that could, in turn, affect the behaviour of the animals. However, for the purposes of this study, we aimed to observe any response specifically to a narrow range of individual, ecologically relevant stimuli, rather than observing the animals’ behaviour in the natural environment. Furthermore, we maintained a constant ambient temperature, which may not be the case in nature due to factors such as winds, waves, or clouds. Further studies may be performed to understand the impact of these factors on the responses of S. roscoffensis to varied stimuli. For example, future work could use sand as a substrate, perform chemosensory experiments under varying temperature and light conditions, or test at different points of the tidal cycle.

Without invalidating their study, the authors must discuss certain points, as no study is perfect.

The method is missing a section on statistical analysis. Without this section, it is not possible to assess the validity and reliability of the results and conclusions drawn by the authors. I will, however, make a few comments based on what is mentioned in the figure titles. Was Dunnet's T3 multiple comparisons test carried out for each time (2, 5, 10 min) separately?
Why did the authors choose these tests? Were the application conditions respected? These details should be included in the method.
Did a single worm do several trials (tyrosine, glutamine, etc.)? If so, were the worms always in the same group? If the groups remain the same, it should be added to the model as a random factor since the analyses are based on repeated measures (One-way repeated measures ANOVA or GLMM).
We thank the reviewer for pointing out this critical omission. We have now added a statistical analysis section to the methods:
Statistical analysis:
All statistical analysis was performed using GraphPad Prism 10. For each trial of each experiment, a fresh set of acoels was used, thus no worms were subjected to multiple stimulation experiments. For time trials, each time point was treated independently. Welch’s test was used to determine unequal variance within the individual groups, and, combined with a relatively low sample size (n=10), Dunnet’s T3 multiple comparison test was chosen. Individual statistical results are presented in the relevant results sections and figure legends.

**Minor issues**

1. I suggest that authors proofread the entire text to correct spelling, grammatical and wording errors. To facilitate correction, authors can read the text starting from the end, or ask a third party who was not involved in drafting to read the text. In addition, certain scientific words relating to animal behaviour are not used correctly. Authors need to revise certain definitions and ensure that the scientific terms used support exactly what they mean.

We thank the reviewer for pointing this out. We have ensured to correct a number of spelling and grammatical errors, including those found in scientific terms

1. Authors will have to modify the formatting of references to meet the journal's requirements :

*References are listed at the end of the manuscript and numbered in the order that they appear in the text. In the text, cite the reference number in square brackets (e.g., “We used the techniques developed by our colleagues [19] to analyze the data”). PLOS uses the numbered citation (citation-sequence) method and first six authors, et al.*

https://journals.plos.org/plosone/s/submission-guidelines#loc-introduction

We have now ensured that the reference style meets the journal requirement

**Abstract (page 2)**

1. In the second sentence, the authors mention that "understanding how distinct sensory cues elicit or modulate certain behaviour thus provides insights into the neuronal adaptations to rapid and continuous changes in the surrounding world". However, there is no reference to neuronal adaptation afterwards, although this seems to be an important subject of the study they way it is mentionned. I consider this paper to be in the field of animal behaviour rather than neuroscience. Authors should be careful about the scope of their claims.

We thank the reviewer for pointing this out, and have removed “neuronal” from the sentence in question to ensure that the scope of the study remains focused.

1. When stating the general problem (lack of knowledge on other sensory systems), I recommend giving a short explanation on why bridging the gap matters, to give more context and justify the purpose of the study.

We thank the reviewer for this suggestion, and have now added a section to the introduction to address this (lines 92-94): Bridging this gap in knowledge may uncover how non-visual sensory modalities govern how S. roscoffensis locates and retains its algal symbiont and selects microhabitats in a highly variable intertidal environment. Establishing these capacities also provides comparative data for the early evolution of multimodal sensory integration in bilaterians.

1. While keeping this section concise with the main result, I suggest going into a little more detail. The method and main results should be incorporated to quickly understand the study when first reading the abstract. I suggest integrating the approach and the main results in a single sentence (e.g. Using XXX method, we found YYY).

Thank you for this suggestion. The end of the abstract now reads (Lines 35-37): Using two-choice and barrier assays, our findings support that S. roscoffensis shows avoidance behaviours to increased temperature and high salinity, preferring cooler environments with lower salinity.

1. What does the main result reveals? The aim of the study seems to be to explore the yet unknown sensory systems of *S. roscoffensis*. What do the results reveal about this? Does it suggest that *S. roscoffensis* can detect chemical and thermal cues?

I suggest adding a final sentence or two about the wider context of the results. For example, but without limitation, authors could mention:

-What the results demonstrate;

-What are the advances/implications in relation to previous works;

- How does the results change the understanding of this field (sensory ecology or neuronal adaptations) / how significant are the results?

Since these additions are likely to increase the size of the abstract, I suggest cutting into the study backgroud, which currently takes up a lot of space compared to the results (i.e. everything before "Currently little is known about...").

Thank you for this suggestion. We have added the following sentence at the end of the abstract:

We demonstrate that early-branching bilaterians possess the sensory capacity to identify specific chemical and environmental stimuli, adding to the knowledge that

1. "is" should be changed for "are" in the following sentence : "When the acoel *Symsagittifera roscoffensis* is exposed…"

We have made this correction, thank you.

1. The study topic is truly interesting and captivating. I think it's worth extending the introduction a little to take the time to properly introduce the concepts and justify the aim of the study.

Thank you for your suggestion, as per the previous comment, we have extended the introduction to include more background and justification for the study.

**Introduction**

1. **Page 3 :** The first paragraph of the introduction should include at least one or two references. An interesting way of inserting them is to add examples to support the facts.

Thank you for this suggestion. We have added a recent reference to the review by Oteiza and Baldwin (2021) to point out that despite the variability the phenotypes are typically conserved (lines 47-49):

While many animals utilise external sensory organs to determine the nature of their surroundings, the compositional, morphological and anatomical organization of sensory organs varies greatly in different animal clades, including different, often unrelated genes being responsible for the same functions, indicating convergent evolution [1]

1. **Page 3 :** In the first paragraph, the authors mention that environmental signals elicit or modulate a behaviour. I suggest to change "behaviour" for "response" or to add nuance (such as "can elicit") as it can be a behavioural or physiological response.

Thank you. We have changed “behaviour” to “response”

1. Through the manuscript, the authors alternate between the terms "signal" and "cue". Authors should be careful to use the correct term, as the two do not have the same meaning. A signal provides intentional (active) information with the aim of influencing the receiver's behaviour. Signals are actively produce to communicate. A cue provides unintentional (passive) information, which may be environmental or behavioural (Freeberg et al., 2021)

We thank the reviewer for this suggestion, and have replaced instances of “signal” to “cue” in order to more accurately reflect the principles.

1. **Page 3 :** In the first sentence of the second paragraph, "thus" should be removed : "The highly variable environmental physical and chemical conditions of intertidal ecosystems thus likely…"

Thank you, this has been removed

1. **Page 3 :** For precision, I suggest that the authors define what they mean by intertidal ecosystems.

Thank you, we have clarified this now (line 55): which are submerged at high tide and exposed to air at low tide,

1. **Page 3 :** "…the direct exposure to light also provides advantages for certain physiological adaptations." The authors should be careful with the wording. It's not the environment that offers an advantage to an adaptation, but the opposite.

Thank you for pointing out this error, indeed the sentence has been changed to read (line 56): certain physiological adaptations also provide advantages to optimise exposure to light

1. **Page 3 :** "*S. roscoffensis* challenges some conventional metabolic principles...". Is it possible to name a few as examples as "metabolic principles" is rather vague.

Thank you for your suggestion, we have added an example of amino acid synthesis as discussed in Boyle and Smith, 1975 (line 67): challenges some conventional metabolic principles such as reliance on the symbiont for amino acid (AA) synthesis [7]

1. **Page 3 :** The following sentence is unclear : "However, despite receiving a range of nutrients and energy from its symbiont (Selosse, 2000), little is known about whether S. roscoffensis requires additional external nutrients (possible mixotrophy) or cues to thrive in this changing environment; and if so, how a hypothetical chemosensory system is used to forage for them."

We have now reworded the sentence into two (lines 71-75): Although the algal symbiont supplies S. roscoffensis with most of its energy and nutrients [9], it is still unclear whether the acoel also needs to obtain extra food from its surroundings (i.e., whether it is partly mixotrophic). If so, it is not yet known what chemical cues are relevant or how a chemosensory system might guide it to those external resources

I understand that the authors question the possible use of additional nutrients and additionnal cues. If this is the case, there has been no mention yet of using any kind of cue. At the beginning of the paragraph, exposure to direct light was discussed, but no clear link was made with the worm and its symbiosis with an algae.

I suggest changing the order of presentation of the concepts in the paragraph to clarify the idea supported.

We thank the reviewer for this comment. We chose not to speculate on any potential external cues as there is currently no known information on whether this phenomenon exists in the acoel. It is the goal of this study to uncover first if sensing is present, to which specific compounds, with the goal of further studies integrating these findings as background for investigating potential food sources. Thus, we simply hypothesise that additional cues may be required. We believe that in addition with the reviewer’s previous comment to clarify the wording of the role of the symbiont adequately addresses this question

1. **Page 4 :** The following sentence is unclear : "While lacking obvious or canonical chemosensory organs (only candidates: (Bery et al., 2010; Sakagami et al., 2024; Todt andTyler, 2007)), *S. roscoffensis* possesses light-sensitive rhabdomeric eyes and a gravity-sensing statocyst (Bailly et al., 2014; Lanfranchi, 1990; Sprecher et al., 2015; Yamasu, 1991)."

What are obvious and canonical chemosensory organs? I think that some examples or a definition would be beneficial to understanding. In the same vein, authors should include a definition or the utility of light-sensitive rhabdomeric eyes and gravity-sensing statocyst.

We thank the reviewer for pointing out that additional context would be useful. We have therefore defined canonical chemosensory organs added functional definitions of rhabdomeric eyes and the statocyst (lines 79-86):

In many animals, chemosensation is mediated by specialised external organs such as insect antennae, vertebrate olfactory epithelia, the antennules and aesthetascs of crustaceans, or the amphid sensilla of nematodes [1]. These organs bear high densities of receptor neurons exposed to the medium and thus are considered “canonical” chemosensory structures. By contrast, no such obvious external chemosensory organs have been described in S. roscoffensis, except a some suggested through ultrastructure analysis, as well as some putative receptor genes .

Like other acoels, the S. roscoffensis possesses paired rhabdomeric eyes: cup‑shaped invaginations lined with microvillar photoreceptors that detect light direction and intensity; and a statocyst, an internal, fluid‑filled vesicle containing a statolith whose deflection under gravity provides the animal with information on body orientation

1. **Page 4 :** As with the abstract, the introduction misses details on the importance of bridging the knowledge gap. Why should we know more about the sensory system of *S. roscoffensis*? Why is this information important to the authors' field of study?

We have now addressed this point both in the abstract as well as in the introduction:

Lines 37-40: We demonstrate that early-branching bilaterians possess the sensory capacity to identify specific chemical and environmental stimuli, adding to the knowledge that may prove useful in understanding marine ecosystems in a period of global climate change that greatly affects aquatic environments.

Lines 91-95: Bridging this gap in knowledge may uncover how non-visual sensory modalities govern how S. roscoffensis locates and retains its algal symbiont and selects microhabitats in a highly variable intertidal environment. Establishing these capacities also provides comparative data for the early evolution of multimodal sensory integration in bilaterians.

1. **Page 4 :** "and" should be changed for "in", and the comma between interdidal and environment should be removed in the following sentence : "Given that this worm lives and aquatic, intertidal, environment…".

Thank you, this has been corrected.

1. **Page 4 :** The authors hypothesize that *S. roscoffensis* should be able to respond to chemical cues from their symbiont and free-living relatives. This is the first mention of cues emitted by the symbiont. This fact should have been mentioned before. I suggest that authors make sure the story told in the introduction leads to the problematics to be investigated and to the aim, and that all this is clearly related to the hypotheses. The research problem/question, hypotheses and predictions must be coherent and aligned.

We thank the reviewer for pointing out this omission, we have now added the context that the method by which the symbiont is located is not known (line 62-63): Tetraselmis convolutae [2], which it locates at the juvenile stage through unknown means and..

**Results**

1. Authors must choose their spelling of behaviour. Sometimes it is spelled with a "u" and sometimes without.

All language has been changed to British English

1. **Page 5 :** This sentence is unclear : "Other neurotransmitter AAs such as glycine, or neurotransmitter

Thank you for pointing this out, the sentence now reads (line 120): Other AAs that act as neurotransmitters such as glycine...

precursor AAs such as tyrosine (dopamine and noradrenaline precursors), tryptophan (serotonin precursor), were not tested for in previous studies."

Was not tested for what?

This sentence now reads: were not tested in regards to navigational response in previous studies

1. The verb "to sense" if often use (e.g. : " Hereby, we tested whether S. roscoffensis is able to sense and respond to the presence of these AA"). The use of this term seems incorrect as it refers to a feeling. I suggest replacing it with the verb "to detect".

Instances of “sense” have been changed to “detect”, thank you

1. The link between the use and the detection of AAs is unclear to me. What use is it to worms to detect AAs that can act as neurotransmitters? Is it an essential nutrient that should be present in their diet? If it's the symbiont that produces AAs for the worm, why should the worm be able to detect them in the environment? Is the aim of this experiment to see whether worms can detect AAs or to check whether glycine, tyrosine and tryptophan are used by worms?

This is an interesting point, for which we know very little in invertebrates. In fact, yes, amino acids are regularly found in seawater, though typically at low concentrations (the most common: glycine, alanine, serine and glutamic acid), coming from different sources like phytoplankton exudates, bacterial metabolism, zooplankton excretion or decomposition of organic matter. Some invertebrates take amino acids directly from the sea water (we don’t know if this applies to S. roscoffensis, but it has never been excluded). But most interestingly amino acids can also act as chemoattractants, influencing behavior of different marine animals (among them several inveretebrates). The scope of this study is not to speculate on the role or effect of these AAs on the acoel, but only to measure any response to the environmental presence. Thus, with the reviewer’s previously addressed comment (‘detect and respond to presence of these AAs’), and the addition of clarification that these are not known to be provided by the symbiont (lines 126-127): ‘as there is no evidence that tryptophan, tyrosine, and glycine are provided by the symbiont’, we believe that it has been made clear that only the response to the presence of the AAs is in question. No claim about consumption or utilisation is being made.

1. The authors alternate between AA and amino acids. The use of one or the other must be constant.

Thank you, we have ensured to define AA as amino acids in the first instance and used AA from then on.

The presentation of the results is not indicative (e.g. PI=-0.1, p=0.74). Many details are missing. These details reinforce the quality and rigor of the results, making the study much more robust. Since the method isn't presented first, (PI) should be placed in brackets as soon as *preference indices* is first mentioned in the text. It should also be mentioned that the PI value corresponds to the mean PI (from what I understood).
I recommend keeping the PI value and the statistical test separate, as they are two different things. The PI should be accompanied by its ± SEM or SD (mean = -0.001, SEM = 0.002) or (mean ± SEM : -0.001 ± 0.002)
The test from which these results come must be mentioned.
The test statistic and its value, the degrees of freedom (or sample size, according to the test) and the p-value must be mentioned. The p-value means nothing without the value of the test statistics and the ± SEM or SD. I recommend reading Amrhein et al. 2019.

Following these suggestions, the presentation would look like this, depending on the statistical test used :

(PI = -0.001, SEM = XX; Brown-Forsythe ANOVA : *F*df1, df2 = XX, p = 0.9996)

We thank the reviewer for this suggestion and have now adjusted all statistical descriptions as recommended

Authors should also ensure that the presentation of results is consistent. Several variants are currently used :

(10 min,PI=-0.22, p=0.09)

(mean preference index -0.45, p<0.0001 vs control)

(PI=-0.001, p=0.9996)

Thank you for pointing this out. These have now all been formatted the same

1. The use of the terms timepoint and timescale should be consistent, with only one of them retained throughout the text. X-minute timepoint is more precise. By using the latter, it is no longer necessary to mention the time in brackets with the results.

Thank you for pointing this out. We have now removed the use of ‘timescale’ and also removed the timepoints from the statistical descriptions

1. The authors should be careful with the wording of this sentence, there are many unfinished ideas :

**Page 6 :** Considering the dynamic nature with varying salinity in intertidal waters due to evaporation and temperature changes during low tides, it has been shown that during low tides the salinity of sea water can change by up to 300% (Geng et al., 2016), we then asked whether changes in salinity of the substrate would result in a preference phenotype.

What is a preference phenotype? This seems a bit out of nowhere as the main goal of the study is to investigate the chemosensory and thermosensory sensitivity of *S. roscoffensis*. I suggest defining the term and making a clearer link with the main objective of the study.

We thank the reviewer for pointing this out. We have now adjusted the relevant section to read as follows (lines 145-147):

can change by up to 300% [24]. Since this is directly related to the environment of the acoel, we hypothesised that these animals may seek specific salinity levels, suggesting an ability to sense this modality. Therefore, we asked whether changes

1. **Page 7 :** For the barrier-assay, the authors should present the results in the same way as before, i.e. the average percentage of crossing with the error, and the result of the statistical tests. Idem for algae tests.

We have now included the relevant statistics in the results, thank you

1. **Page 7 :** For the barrier-assay, how the authors determined that this was an aversion and not some other physiological effect? What would have been the observed results if the cause of non-crossing was, for example, reduced mobility?

Thank you for pointing out this lack of clarity. We indeed considered this, and monitored the behaviour to ensure that no animals were ‘stuck’ in the barrier zone, which we did not observe. Nonetheless, we used 10 minutes as the experimental length to negate any effect of low mobility, as this amount of time is overwhelmingly higher than is required for the animals to cross 1cm (typically seconds). We have clarified this in the methods (lines 281-283): with the attempts and successful crosses counted. 10 minutes were given for each trial in order to negate any effects of reduced mobility, which was not noticed by the experimenter.

1. **Page 8 :** "These experiments support the notion that the animals…". "Animal" should be changed for the species name. This should be done throughout the text to ensure clarity.

All reference to *S. roscoffensis* have now been adjusted to the species name and not the word animal

1. **Page 8 :** "This in turn suggests that while low temperatures are neither attractive nor aversive, high temperature is decidedly an aversive cue." Why can't it be a preference for lower temperatures rather than an avoidance of higher ones? How did the authors come to this interpretation?

Thank you for pointing this out. We came to this conclusion since the animals showed no difference in preference between the low and ambient conditions, suggesting that below a certain threshold they do not display a preference for increasingly lower temperatures. Therefore, the negative preference must be due to high temperature. We have clarified this in the text (lines 228-230): high temperature is decidedly an aversive cue, since animals show no preference between ambient and low temperatures, and only negative preference to high temperature.

**Discussion**

1. **Page 9 :** "Here we analysed the preferences to chemical and physical cues of the acoel flatworm Symsagittifera roscoffensis…" The aim in the introduction should be the same as the one mentioned in the discussion.

We have now clarified this (line 328-329): Here we investigated sensory capabilities in relation to chemical and physical cues of the acoel flatworm Symsagittifera roscoffensis

1. Is the aim achieved? Do the results support the hypotheses? To me, these two elements are essential and and fundamental to rigorous scientific work. Yet this is missing.

We have pointed out that the hypothesised sensing was observed in lines 331-333:

our findings indicate that the acoel Symsagittifera roscoffensis can display distinct behavioural preferences when exposed to a range of chemical and environmental stimuli

1. **Page 9 :** "it is logical that the animals would prefer a specific level of salinity to remain in an isotonic environment and avoid a hypotonic one."

Why is this logical? If these worms are used to constant and drastic changes, isn't their physiology adapted to this? Wouldn't they be able, by homeostasis for instance, to survive in different salinity levels without needing to move? Are they able to move great distances (I imagine that a movement of 87 mm, the size of the petri, in nature does not really change the salinity of the environment)? Could it be a behaviour related to foraging, reproduction or feeding? Do the authors have any references to back up their claims?

Thank you for pointing this out, we have now reworded this to read “it may be presumed that...” (line 336), while in the next sentences we address why this does not appear to be the case.

1. **Page 10 :** Could the aversive response be due to the algae taking some control over its host (kind of parasite manupulation, but with a symbiont) (Hughes & Libersat, 2019)?

While this is an interesting idea, this we chose not to speculate on this since there is nothing known about this topic. Indeed, part of our internal logic behind choosing neurotransmitter-related AAs was for this reason, as discussed in the introduction

1. **Page 11 :** "Thus, the “low temperature” condition in our assay may not be sufficiently lower than the temperature experienced by S. roscoffensis in the natural environment." How did the authors select the temperatures and salinity levels? Why not choose the maximum and minimum limits of the natural environment?

Thank you for this comment. Indeed, the temperatures were chosen mainly with consideration to having enough realistic difference between conditions with practical considerations in mind. Since it is very difficult to estimate what the animals may be naturally subjected to (and, to our knowledge, no such studies have been performed), we chose a reasonable low (5c) and a reasonable high (30c) which the animals would experience mainly during low tide (thus, subject to atmospheric temperature rather than sea temperature). We have clarified this in the methods (lines 297-298): The temperatures were selected to ensure sufficient difference between conditions under practical limitations.

1. **Page 11 :** The last sentence is hard to understand and rather vague. I suggest rephrasing and that the link between the final conclusion and the rest of the study be more thoughtful.

Thank you, we have rephrased the final sentence to read: In all, we show that early-branching bilaterians display sensory responses to a variety of stimuli, and further studies can be performed to understand how the natural environment can affect the sensory responses of S. roscoffensis.

**Materials and Methods**

1. There is a lack of detail in the collection and maintenance section to ensure reproducibility. As experiments are performed on live animals, capture and maintenance conditions may influence the animals' behavioural response measured during trials. I recommend that authors add information about:

- GPS coordinates at collection site

We have added the GPS coordinates of the collection sites

- Date of capture (at least month and year)

We have added the collection date

- Lifestage or average size, if known

We have clarified that all animals were adults, however since the size varies greatly between individuals, this was not taken into account

- Sex (M, F or unknown)

We have clarified that animals were not discriminated by sex

- Presence or absence of *Tetraselmis convolutae* symbiont

We have clarified that only animals with visible symbiont presence were used

- Number of worms initially captured, number of worms per plastic container

While we did not specifically count the number of worms in each container, based on the numbers used in experiments we estimate that each container held between 1000-5000 animals. This information has now been included

- Date of trials (How long they acclimated to the lab?)

We have added the information that the earliest experiments were performed 2 days after collection, and the latest 7 days after collection.

- Relevant holding conditions (e.g. temperature, oxygen level). Were conditions constant or dependent on outside weather conditions? Has the water been treated in any way (UV, sand filter, etc.) to prevent infection or parasites?

We have clarified this section to read as follows: ) with filtered natural sea water, continuously flowing to ensure consistent oxygenation, at constant water temperature (14±1°C) and constant air temperature (21°C) at the Station Biologique de Roscoff

- Feeding regime during holding and trials (I imagine this may influence the preference for one side of the agarose and the motivation for foraging).

We have clarified that the animals were not provided supplementary food (lines 246-247): Animals were not provided supplementary food outside of what may have been present in the surrounding water and sand

In order to verify the thinking behind the experimental protocol, I have a few questions. The aim here is not to cast doubt on the experiments, but to explore possible limitations and confounding factors : Was the number of worms per petri dish too high to allow each worm to move freely and voluntarily and to make a real choice? Could the degree of sociability of these worms have influenced the result? How did authors choose the number of worms per petri dish?
The number of worms per dish was specifically chosen to avoid overcrowding without the burden of individually counting the number of worms before each experiment. Approximately 1ml of the worm water was collected for each experiment, resulting in the numbers seen. The counting was performed upon quantification by means of taking photographs of the arenas upon each timepoint (now clarified in the text). We include below sample images of the arenas during trials, in hopes that this satisfies the reviewer’s concern that the arenas were not overcrowded:

What was the temperature in the petri dish during chemical trials? Was it stable? This is important because temperature preference is tested later.
We have clarified that the temperature was kept constant throughout the experiments at ambient 18°C
Was the lighting constant and identical on both sides? Could this have influenced the worms' preference?
While the lighting in the room was uniform, between each individual N the dishes were rotated 180° in order to mitigate any potential effect of light response. This has been clarified in the text (line 261)
Did the AAs and algae remain in the agarose gel or gradually dilute in the water? If diluted gradually, could this have influenced the choice over time? How did the authors ensure that the worms could actually detect the cues in the agarose?
We thank the reviewer for this point, and indeed we considered the same when designing the experiments. While it is true that over time the compounds will dilute out of the agarose, we specifically chose the selected timescale in order to mitigate any effect, and additionally counted all experiments at early (2min) intermediate (5min) and longer term (10min) time points to see if any effects can be observed. If there was significant dilution, the effects would diminish over time, when in fact we see the opposite. It is known that worms do not swim, but instead crawl on the surface using cilia. We did not, and still do not, know how exactly detection happens since the sensory anatomy is unclear, as discussed in the introduction.
Were the experimenters present during the trials? If so, did this influence the worms' response?
The experimenter was in the same position relative to the plates for all experiments, with the rotation of the plates also serving the purpose of mitigating any effect this may have. Anecdotally, we did not observe any effect of distance or position of the experimenter on the responses of the animals, but this data was not collected or quantified.
Did counting during trials disturb the worms? What have you done to reduce the disruption caused by counting? Did you count from a video/image after?
We have now clarified in the methods that counting was done by taking an image at each time point and counting after the experiment. The animals were not disturbed throughout the trials
Did you count manually or with a software? If you counted manually, how did you reduce or limit the biases of the person counting? If you counted with a software, what was its name/brand?
The counting was performed in a blind manner, i.e. the counter was not aware of which experiment, timepoint, or the stimulus side they were counting. This has now been added to the methods (lines 265-266)

1. *S. roscoffensis* were grouped into groups of 50 to 200 individuals. How many trials (number of group) were carried out for each experiment? This should be mention in the method.

Thank you for pointing this out. We performed 10 trials for each experiment, while this was mentioned in the legends and in the results, we have now added this to the methods.

1. Have non-responsive individuals been removed from the count? How many did you remove? What were the criteria for determining that an individual was non-responsive?

Since only animals that showed negative geotaxis were selected (i.e. alive), we did not monitor inactive/slow/sleeping animals, with the intention that the relatively large group size of each trial would mitigate any effects of inactive animals. Anecdotally, we can confirm that the vast majority of the animals were active by the end of each trial, with only a negligible number being inactive or dead. We have clarified this point in the text.

1. If not already done, I strongly suggest that authors read Jutfelt et al. (2017) article on two-current choice flumes for testing avoidance and preference in aquatic animals. The experimental apparatus is not quite the same, but I believe that certain ideas could guide the authors in explaining their method.

Thank you for this suggestion. Since our experimental setup was very different we chose to present the results in the way that is typically done for such an experiment, i.e. larval Drosophila.

Here's a point raised in the article by Jutfelt et al. (2017) : "In any event, sufficient exploration of the arena by the test animal is an obvious prerequisite before attribution of side preferences can occur."

Did the worms have the opportunity to sample the cues on both sides of the petri dishes before the experiment began? If not, how can the authors be sure that a preference was measured if some worms didn't even know what was on the other side?

Thank you for pointing this out. In our experience and in personal communications with experts at Roscoff, we know very well that the animals take at most 1-2 minutes to completely circle or cross the 87mm petri dish. We therefore used the varied timepoints to ensure that animals have sufficient time to explore, and used the 10-minute timepoint for this reason. We have clarified this in the methods.

1. **Page 15 :** Please correct the names of statistical tests : Brown-Forsythe and Welch ANOCA test with Dinnet’s T3 multiple comparisons tested against the control group.

Thank you for pointing out this mistake, we have corrected it alongside others from the previous comment.

1. **Page 16 :** There's an analysis of the results in the title of figure 3 C, which isn't there for the other figures. I think this can be removed.

This has been adjusted, thank you.

1. There is no title for figure 4.

The figures have been uniformly laid out. Thank you.

**References**

Amrhein, V., Greenland, S., & McShane, B. (2019). Scientists rise up against statistical significance. *Nature*, *567*(7748), 305-307. https://doi.org/10.1038/d41586-019-00857-9

Freeberg, T.M., Book, D.L., Jung, H., Kyle, S.C. (2021). Communication, Cues, and Signals. In: Shackelford, T.K., Weekes-Shackelford, V.A. (eds) Encyclopedia of Evolutionary Psychological Science. Springer, Cham. https://doi.org/10.1007/978-3-319-19650-3_2728

Hughes, D. P., & Libersat, F. (2019). Parasite manipulation of host behavior. *Current Biology*, *29*(2), R45-R47. https://doi.org/10.1016/j.cub.2018.12.001

Jutfelt, F., Sundin, J., Raby, G. D., Krång, A., & Clark, T. D. (2017). Two‐current choice flumes for testing avoidance and preference in aquatic animals. *Methods in Ecology and Evolution*, *8*(3), 379-390. https://doi.org/10.1111/2041-210X.12668
